# Atypical Presentation of Flatbush Diabetes During a Febrile Illness: A Case Report and Review of Literature

**DOI:** 10.7759/cureus.72998

**Published:** 2024-11-04

**Authors:** Asif Dabeer Jafri, Srikant K Dhar, Tanmaya Brahmadarshini Bhuyan, Promod K Tudu, Mareya Zeenat, Kayenaat Rizvi

**Affiliations:** 1 Internal Medicine, Sum Ultimate Medicare, Bhubaneswar, IND; 2 Internal Medicine, Institute of Medical Sciences (IMS) and Sum Hospital, Bhubaneswar, IND; 3 General Medicine, Sum Ultimate Medicare, Bhubaneswar, IND; 4 Pharmacology, Era's Lucknow Medical College and Hospital, Lucknow, IND

**Keywords:** diabetic keto acidosis, flatbush diabetes, glycated hemoglobin (hba1c), maturity onset diabetes of the young, type 1 and type 2 diabetes mellitus

## Abstract

Flatbush diabetes is a significant clinical condition that is gaining recognition on a global scale. Although it's commonly believed that individuals who exhibit symptoms of diabetic ketoacidosis (DKA) have type 1 diabetes and need to take insulin continuously for the rest of their lives, this isn't always the case. Understanding the differences between the natural histories of type 1 and 2 diabetes mellitus (DM) and Flatbush diabetes will aid in early diagnosis and appropriate management. We present a very unusual case of Flatbush diabetes in a young male who presents with recurrent episodes of DKA during febrile illness. Only a few such cases have been documented in the medical literature, despite recent advancements in diabetes research.

## Introduction

Ketone-prone diabetes (KPD), commonly referred to as atypical or Flatbush diabetes, predominantly affects middle-aged, overweight males who typically have a significant family history of type 2 diabetes [1]. The primary features of KPD, a newly characterized type of diabetes mellitus (DM), include spontaneous ketoacidosis in the absence of autoantibodies and β-cell dysfunction [2]. While the underlying pathophysiological mechanisms and etiology remain unclear, initial evidence suggests that individuals with type 2 diabetes who are susceptible to ketosis may exhibit a unique vulnerability to glucose desensitization [3]. At the time of initial diagnosis, both insulin action and secretion are impaired. By initiating proactive diabetes management early, it is possible to markedly improve β-cell function as well as insulin sensitivity, possibly allowing the therapy to be stopped after a few months.

## Case presentation

A male patient, aged 20, was brought to our hospital from another medical facility after being diagnosed with type 1 DM. He presented with the chief complaints of pain in the abdomen and vomiting for one day. He had no past medical history of any chronic illness nor any family history of diabetes. He had a history of recurrent diabetic ketoacidosis (DKA). On examination, he was conscious, oriented, and febrile (38.5°C). His vitals at the time of admission were as follows (heart rate 124/min, respiratory rate 28/min, blood pressure 100/70 mmHg, and oxygen saturation of 94% on room air). A diagnosis of DKA was established due to high blood glucose levels, the presence of metabolic acidosis as indicated by arterial blood gas analysis (pH 7.25, partial pressure of carbon dioxide (pCO2) 30, partial pressure of oxygen (pO2) 58, and bicarbonate (HCO3) 13.9, lactate 1.5), and a positive result for urinary ketones. He was managed with intravenous insulin, intravenous (IV) fluids, and antibiotics, along with other symptomatic and supportive care. After receiving the appropriate treatment, his blood glucose, ketones, and anion gap returned to normal. He stayed in the hospital for 14 days, where he was managed with subcutaneous insulin, which was subsequently discontinued due to the achievement of euglycemia. To mitigate the possible immediate effects of glucose toxicity or desensitization on β-cell function, an evaluation of β-cell secretory activity and β-cell autoimmunity was conducted after the complete resolution of diabetic ketoacidosis. This evaluation was performed after four weeks, accompanied by continuous glucose monitoring. The findings indicated an average glucose level of 74 mg/dl, a glucose management indicator of 5.1%, and a glucose variability of 25.9. Blood and urine cultures were performed to determine the underlying cause of the fever, but no growth was detected. Additionally, an MRI of the abdomen was carried out in response to persistent abdominal pain, which indicated no abnormalities. Genetic testing for maturity-onset diabetes of the young (MODY) was negative, and his echocardiography showed no abnormalities. The details of the laboratory parameters, which include complete blood count, liver function tests, kidney function tests, thyroid profile, insulin antibodies, C-peptide, pancreatic enzymes, viral markers, and complement level (C3), are presented in Table 1. 

**Table 1:** Blood parameters ordered during present admission. HB: hemoglobin; TLC: total leucocyte count; TPC: total platelets count; S. Na: serum sodium; S. K: serum potassium; S. Cl: serum chloride;  SGOT: serum glutamic oxaloacetic transaminase; SGPT: serum glutamate pyruvate transaminase; TSH: thyroid stimulating antibody; HBA1c: glycosylated hemoglobin; Anti GAD 65: glutamic acid decarboxylase antibody; IA-2 antibody: islet antigen 2 antibody; Urine R/E: urine routine examination

Analyte	Patient result	Reference value
HB	13.3	13-17 g/dl
TLC	13.850	4-11 x 10^9^/L
TPC	234	150-400 x 10^9^/ L
S. Na	137	135-145 mEq/ L
S.K	3.8	3.5-5 mEq/L
S.Cl	1O1	96-115
SGOT	15	5-40 U/L
SGPT	10.7	5-40 U/L
T. Bilirubin	0.89	0.0- 2.0 mg/dl
Alkaline phosphatase	226	40-129 IU/L
T. Protein	6.49	6.0-8.3 gm/dl
S. Albumin	4.29	3.3-5.2 mg/dl
Urea	20	13-45 mg/dl
Creatinine	1.0	0.5-1.5mg/dl
Calcium	8.4	8.6-10.3 mg/dl
TSH	1.49	0.270-4.20
T3	57.55	84.63-201.08
T4	8.18	5.13-14.06
HBA1c	5.5	4.8-5.9
C-Peptide	2.22	0.1-5.19 ng/ml
C3	167	75-135 mg/dl
Anti-GAD antibody	0.65	<5 unit/ml
IA-2 antibody	0.25U/ml	Negative:<7.5 U/ml
Procalcitonin	0.78	<5 ng/ml
S. Lipase	12.7	13-60
S. Amylase	32	<96
CRP	<0.1	0.00-1.00
HIV	Negative	
HCV	Negative	
HBsAg	Negative	
Urine R/E	Pus cells 5, Sugar 4+, Protein +, Ketone bodies 2+	Pus cell 0-5/hpf, sugar/protein/ketones-nil

Two years ago, he experienced two episodes of DKA during his febrile illness, both of which were successfully managed. After resolution, he was treated with insulin for two months, during which his fasting and postprandial blood sugar levels returned to normal, eliminating the need for further insulin. During that period, he underwent an evaluation for diabetes, and the results of his tests, which included hemoglobin A1C (HbA1c) (5.4), C-peptide, anti-GAD, and anti-insulin antibodies, were all found to be within normal limits.

On the current admission, he received an IV insulin infusion, IV fluids, and antibiotics (ceftriaxone). Insulin infusion was given at the rate of 0.1U/kg/hour, along with an infusion of potassium chloride. His DKA resolved within 48 hours of hospital admission. Following the restoration of his normal appetite and hemodynamic stability, he was transitioned back to subcutaneous insulin. By the fifth day, his blood sugar levels normalized, resulting in no further insulin administration. Throughout his hospital stay, DKA was effectively managed, and no adverse reactions to the treatment were observed. Upon follow-up at three months, his C-peptide, HbA1c, fasting, and post-prandial blood sugar levels were all within the normal limits.

## Discussion

The development of DKA in people who do not fit the typical criteria for type 1 or type 2 DM is a characteristic of Flatbush diabetes, which is recognized as a heterogeneous illness [4,5]. These patients had impaired insulin secretion and action at presentation, but with intensive diabetes management, β-cell activity and sensitivity of insulin are significantly improved, allowing treatment to be stopped for insulin within a few months [6,7]. A case series comprising adult overweight Afro-Caribbean patients with a diagnosis of DKA was carried out in 1994 by Banerji et al.; during this time, the term "Flatbush diabetes" began to emerge in the medical literature to refer to the area of New York where these patients lived [8]. Since the mid-1990s, there has been a rise in the number of individuals diagnosed with DKA who do not necessitate long-term insulin therapy. These individuals' circumstances are similar to type 2 DM since a sizable majority of them are obese and have a family history of the disease. In the wake of these findings, novel classification systems for diabetes have been established. Four distinct classification schemes have been created to categorize patients with KPD into clinically significant and relevant subgroups: The Aβ system, a revised American Diabetes Association (ADA) classification, a BMI-based system, and the ADA classification.

The Aβ system, where A denotes autoantibody status and β signifies β-cell functional reserve, serves as the most dependable classification framework for predicting glycemic control and the necessity for insulin therapy one year after the onset of diabetic ketoacidosis. This system categorizes individuals with diabetes into four distinct groups: 1) A+β-: Autoantibodies present, β-cell function absent; they need to be treated with exogenous insulin for life. This variant is also known as KPD type 1A, often known as type 1A diabetes; 2) A-β-: Autoantibodies absent, β-cell function absent; they need to be treated with exogenous insulin for life. This variant is also known as KPD type 1B, often known as type 1B diabetes; 3) A+β+: Autoantibodies present, β-cell function present; over time, these individuals lose their β-cell reserve and need to be on exogenous insulin therapy forever. This variant is also known as KPD type 2A; 4) A-β+: Autoantibodies absent, β-cell function present; most people (especially those with recent onset) can stop using exogenous insulin therapy and can be long-term treated with oral hypoglycemic medications or diet alone. This variant is also known as Flatbush DM or KPD type 2B.

While A+β- and A-β- patients differ in terms of immunology and genetics, they share the clinical features of type 1 diabetes, which have extremely lower β-cell function. In contrast, A+β+ and A-β+ patients also differ in terms of immunology and genetics but share the type 2 diabetes clinical features with preserved β-cell functional reserve. Uncertainty surrounds the precise incidence and prevalence of ketone-prone diabetes. Reports make it very clear that this kind of diabetes is not as common as thought and is not well documented. Due to their lower prevalence, Asian and White populations may account for less than 10% of people presenting with DKA [9,10]. Genetic variables, viral infections, and other metabolic factors like oxidative stress have all been linked as a triggering mechanism for DKA in Flatbush diabetes. Following the first occurrence of DKA in African American patients with the A-β+ variant, Umpierrez et al. investigated the contributions of glucotoxicity and lipotoxicity to the development of significant yet partially reversible impairment in β-cell function [11]. It was discovered that acute hyperglycemia significantly reduced the C-peptide response to glucose stimulation, which helped to explain the A-β+ subtype's reversible β-cell failure [12].

Patients with Flatbush diabetes have a phenotypic profile that differs significantly from those of type 1 DM. Most individuals diagnosed with KPD are typically middle-aged, around 40 years old, and exhibit obesity, frequently presenting with unprovoked DKA. In contrast, the presentation of our case is atypical, as the individual is comparatively young and has a lean body composition with no family history of diabetes. Additionally, he experiences recurrent episodes of DKA, despite maintaining an HbA1c level consistently below 5.7. This deviates from the classical characteristics typically associated with Flatbush diabetes. The frequency of males with ketosis-prone type 2 diabetes seems to be unaffected by age or the level of obesity at presentation. MODY is a monogenic disorder resulting from a mutation in a single gene, bears similarities to Flatbush diabetes and accounts for less than 5% of all diabetes cases, frequently being misdiagnosed as type 1 or type 2 diabetes mellitus. It is characterized by non-ketotic hyperglycemia, with individuals typically lacking pancreatic autoantibodies and often presenting a positive family history. Furthermore, there may be involvement of additional organ systems, such as the kidneys, liver, and intestines [13,14]. However, in our case, a negative family history, negative genetic testing for MODY, and the absence of involvement of extrapancreatic organs can effectively rule out this diagnosis. In a study carried out by Umipierres et al., the average laboratory results of patients with KPD were found to be similar to our laboratory values. The remission phase may persist for several years, occurring sporadically between episodes of unexplained and spontaneous ketosis and hyperglycemia, with HbA1c levels remaining below 6.4, similar to our case [15].

According to research by Umpierrez et al., two-thirds of KPD type 2 diabetes patients who received diet-only care returned to hyperglycemia after two years, as happened in our case [16]. It was also demonstrated that the use of metformin or sulphonylurea for treatment was beneficial in extending the length of normoglycemic remission and avoiding ketoacidosis [17]. Long-term management involves measurements of autoimmunity and β-cell functional reserve that should be made four to eight weeks following the resolution of ketoacidosis. Pancreatic β-cell function is evaluated by measuring C-peptide levels, C-peptide response to glucagon, and fasting blood glucose. C-peptide levels serve as established indicators of the reserve capacity of pancreatic β-cells [18]. Previous studies have indicated that the simultaneous testing of GAD65 autoantibodies and IA2 autoantibodies yields a sensitivity of as high as 98% and a specificity ranging from 98% to 100% for diagnosing type 1 diabetes [19]. Since the autoimmune status of a patient predicts their long-term need for insulin, autoimmunity against β-cells must also be evaluated. The mechanism underlying diabetes that is susceptible to ketosis is still unclear and needs more research.

The implementation of insulin therapy and the subsequent achievement of normoglycemia have been associated with notable and rapid improvements in β-cell function among these patients, akin to the case presented here. A comprehensive evaluation involving the endocrinologists led to the identification of KPD. This diagnosis was based on the initial observation of reduced insulin secretion, which was later followed by a recovery of both insulin sensitivity and secretion once the acute phase had subsided. Our patient meets the diagnostic criteria for Flatbush diabetes as there was no evidence of β-cell autoimmunity and normal function of β-cells (A-β+). This conclusion is supported by the absence of antibodies and the presence of measurable C-peptide levels.

Financial limitations prevented us from doing a full genomic investigation to identify any genetic mutations or predisposing polymorphisms.

## Conclusions

Flatbush diabetes should be taken into account for any individual exhibiting ketosis, regardless of whether there is a family history of diabetes, provided that autoantibodies are absent and β-cell function remains intact. Significant improvement in glycemic parameters is observed after the resolution of glucotoxicity. This acute impairment is transient, and β-cell activity recovers robustly afterwards. The management of Flatbush diabetes is not the same as for type 1 and 2 diabetes. This insight may facilitate early cessation of treatment, as dietary management alone could prove effective for these individuals.
